# In Memoriam Geoffrey Burnstock: Creator of Purinergic Signaling

**DOI:** 10.1093/function/zqaa006

**Published:** 2020-06-25

**Authors:** Alexei Verkhratsky, Herbert Zimmermann, Maria P Abbracchio, Peter Illes, Francesco DiVirgilio

**Affiliations:** 1 Faculty of Biology, Medicine and Health, The University of Manchester, Manchester, M13 9PT, UK; 2 Achucarro Centre for Neuroscience, IKERBASQUE, 48011 Bilbao, Spain; 3 Institute of Cell Biology and Neuroscience, Molecular and Cellular Neurobiology, Goethe-University, Frankfurt am Main, Germany; 4 Department of Pharmacological Sciences, Laboratory of Molecular and Cellular Pharmacology of Purinergic Transmission, University of Milan, Milan, Italy; 5 Rudolf Boehm Institute for Pharmacology and Toxicology, University of Leipzig, Germany; 6 Department of Medical Sciences, University of Ferrara, Ferrara, Leipzig, Italy

**Keywords:** ATP, purinergic signaling purinoceptors, vesicular release, ectonucleotidases, inflammation

## Abstract

Geoff Burnstock (1929–2020) discovered purinergic signaling in a fastidious research that started in early 1960 and culminated in a concept of purinergic nerves in 1972. Subsequently, Geoff developed the concept of purinergic transmission and demonstrated ATP storage, release, and degradation in the context of cotransmission, which was another fundamental concept developed by him. Purinergic transmission contributes to the most fundamental physiological functions such as sensory transduction, regulation of heart rate, smooth muscle contraction, bile secretion, endocrine regulation, immune responses, as well as to various pathophysiological conditions, including inflammation, cancer, neuropathic pain, diabetes, and kidney failure.

I would like to remind the reader of the extraordinary influence of fashionable concepts in science. Gifted and meticulous workers will perform remarkable contortions to fit their data into accepted dogma, especially if established by powerful and brilliant personalities at the forefront of the field. They will often dismiss or ignore data that fall outside interpretation by current theory, searching hard for technical or artefactual explanations. Once a new attitude becomes acceptable, then the same data can be miraculously redeployed to support it.

Geoffrey Burnstock^1^

Geoffrey Burnstock’s (Figure 1) journey of life came to a sad end on June 3, 2020 but what a journey it was! Geoff was born in 1929, which was also the year of birth of ATP, the molecule of life that was at the very core of Geoff’s academic career. ATP was discovered almost simultaneously in the United States and Germany; Cyrus Hartwell Fiske and Yellagaprada SubbaRow were the first to describe ATP in Harvard, probably as early as in 1926. Nonetheless, Karl Lohmann who did similar experiments in Berlin (under the imaginative supervision of Otto Meyerhoff) was first to publish. Lohmann paper appeared in *Naturwissenschaften* in August,^2^ whereas Fiske and SubbaRow managed to have their story printed in *Science* in October 1929.^3^ Very soon Fritz Lipman introduced the concept of the “high-energy phosphate bond”^4^ and ATP became firmly associated with cell energetics. The year 1929 also witnessed the seminal discovery of Alan Drury and Albert Szent-Györgyi von Nagyrapolt who found that a nucleoside adenosine and adenylic acid (adenosine-5'-monophosphate, 5'-AMP) act as signaling molecules in the cardiovascular system.^5^ Thus, the set for Geoff’s scientific life was set.

**Figure 1.**
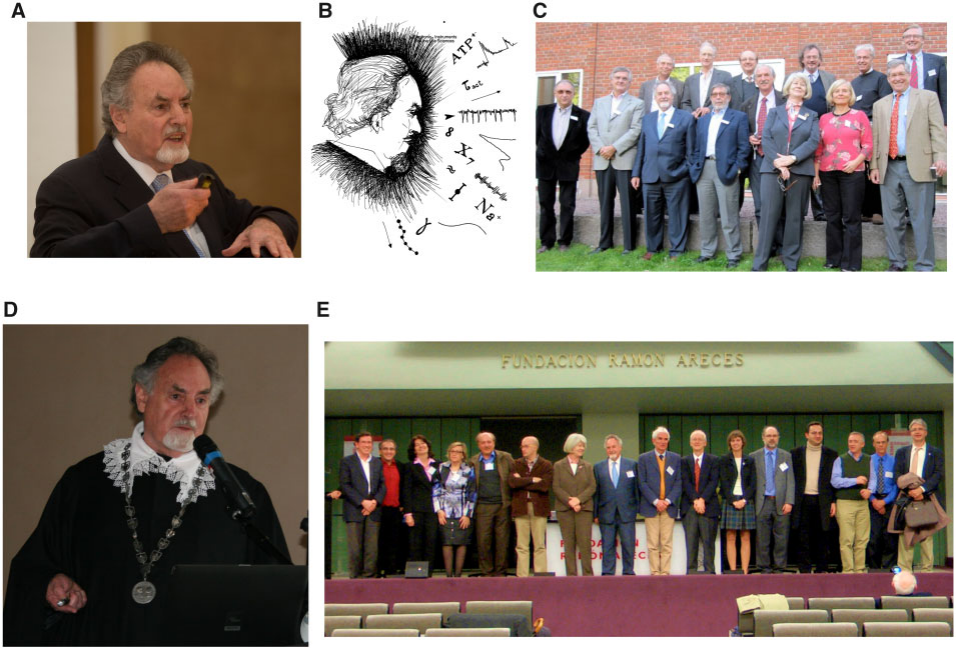
Geoffrey Burnstock (1929–2020). (**A**) Geoff Burnstock with his typical enthusiasm when presenting science at the occasion of the award of the Doctor Honoris Causa at the Goethe University Frankfurt in 2007. (**B**) Geoff portrait was drawn by Dr. Davide Ferrari from Ferrara. (**C**) Celebrating 80th birthday of Geoff and ATP in Karolinska Institute, Nobel Forum, Stockholm, Sweden, September 18–19, 2009. First row (from the left): Oleg Krishtal, Alan North, Geoffrey Burnstock, Alexandre Ribeiro, Michail Sitkovsky, Maria-Teresa Miras-Portugal, Maiken Nedergaard, Bruce Cronstein. Second row: Bertil Fredholm, Kendall Harden, Herbert Zimmermann, Alexei Verkhratsky, Helmut Kettenmann, Jürgen Schrader. (**D**) Geoff in Ferrara, on June 4, 2009 being awarded the Copernicus Gold Medal of the University of Ferrara. (**E**) Geoff at the meeting on “Extracellular Nucleotides and P2 Receptors: Relevance in Physiology and Therapeutic Applications” in Madrid 2011. From left to right: Antonio R. Artalejo, Alfonso Araque, Beatrize López-Corcuera, Esmerilda García-Delicado, Herbert Zimmermann, Jesús Pintor Just, Maria Teresa Miras-Portugal, Geoffrey Burnstock, Francesco Di Virgilio, Simon C. Robson, Beata Sperlagh, Carles Solsona, Miguel Díaz-Hernández, Lisardo Bosca, Douglas Fields, Antonio Cuadrado.

## ATP as a Neurotransmitter: Fighting Against the Odds

“Of known natural processes that might pass on excitation, only two are, in my opinion, worth talking about. Either there exists at the boundary of the contractile substance a stimulative secretion in the form of a thin layer of ammonia, lactic acid, or some other powerful stimulatory substance, or the phenomenon is electrical in nature”.^6^ This sentence written by Emil Du Bois-Reymond in 1877 opened the era of intercellular signaling mediated by molecules moving between cells through gap junctions or by chemical transmitters secreted by the cells and acting on specific sites located on the plasma membrane. The synapse (*syn*, σύν meaning together and *haptein* ἅπτειν meaning clasp) as a structure connecting nerve cells was defined by Michael Foster and Charles Scott Sherrington in 1897.^7^ The principles of chemical neurotransmission were laid down by John Newport Langley and Thomas Renton Elliott in the early years of the 20th century. Elliot suggested that epinephrine can be a neurotransmitter; while Langley defined neurotransmitter receptors as “receptive substances… capable of receiving and transmitting stimuli of target cells”.^8–10^ The first neurotransmitter acting through diffusion and binding to specific receptors was acetylcholine discovered by Otto Loewi.^11^ Noradrenaline was experimentally proven to act as a neurotransmitter in 1946^12^; γ-aminobutyric acid (GABA) and glycine were identified and accepted as *bona fide* inhibitory neurotransmitters in the 1970s,^13–15^ while it took about 20 years for glutamate to be included into the class of neurotransmitters.^16^^,^^17^

**Figure 2.**
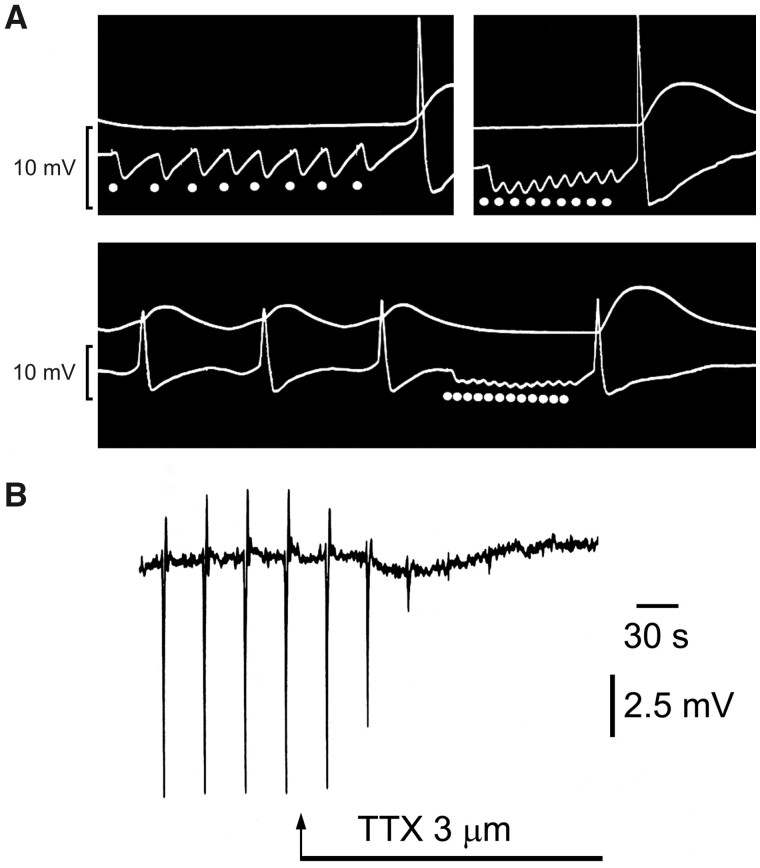
NANC Neurotransmission. (**A**) Sucrose gap records from smooth muscle of guinea pig taenia coli showing hyperpolarizations in response to different stimulation frequencies (1, 3, and 5 Hz) of intrinsic nerves in the presence of atropine and guanethidine. (**B**) Sucrose gap recording of membrane potential changes in smooth muscle of guinea pig taenia coli in the presence of atropine (0.3 µM) and guanethidine (4 µM). Transmural field stimulation (0.5 ms, 0.033 Hz, 8 V) evoked transient hyperpolarizations, which were followed by rebound depolarizations. Tetrodotoxin (TTX, 3 µM) added to the superfusing Krebs solution (applied at arrow) rapidly abolished the response to transmural field stimulation establishing these as inhibitory junction potentials in response to NANC neurotransmission. Figure is reproduced with permission from Burnstock.^18^

**Figure 3.**
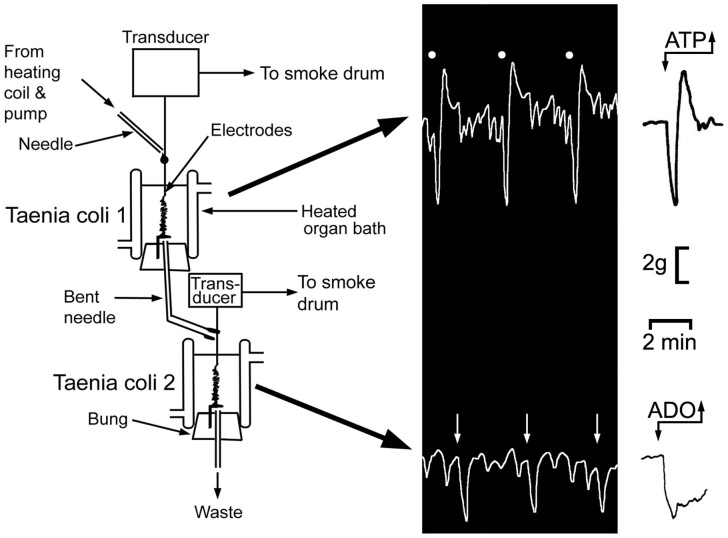
The Loewi-Inspired Experiments Carried out by Geoff Burnstock and Anny Smythe in 1966. The upper guinea pig taenia coli innervated preparation was stimulated at 5 Hz for 40 s every 6 min at 50 V and 2 ms duration, in the presence of atropine and guanethidine to elicit typical NANC responses, fast relaxation followed by rebound contraction. The perfusate passed over the lower taenia coli preparation to produce slow relaxations, but not followed by rebound contractions. In later experiments, we showed that, while the response of the taenia coli in the upper chamber was mimicked by ATP, the response in the lower chamber was mimicked by adenosine, the ATP released from the upper preparation being hydrolyzed rapidly by ectonucleotidases to adenosine before reaching the lower preparation. Figure is reproduced with permission from Burnstock and Verkhratsky.^23^

**Figure 4.**
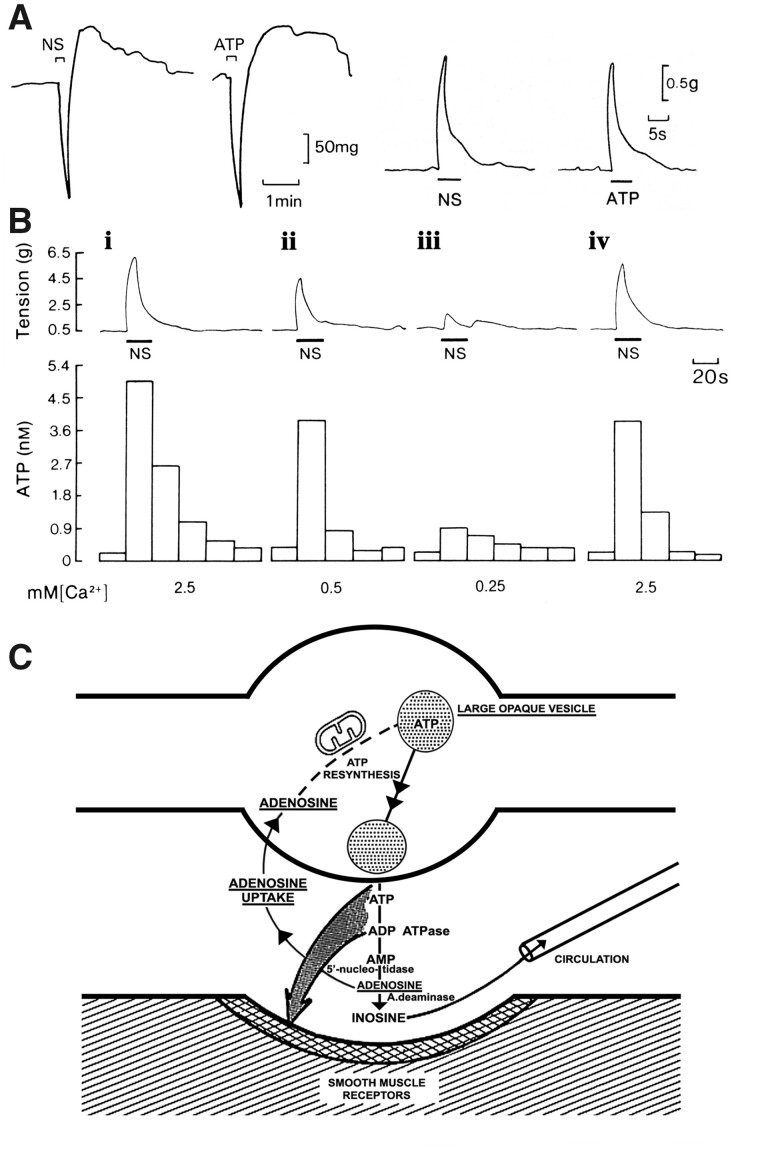
Evidence for ATP as a NANC Neurotransmitter. (**A**) Left-hand side: responses of the guinea pig taenia coli to NANC nerve stimulation (NS, 1 Hz, 0.5 ms pulse duration, for 10 s at supramaximal voltage) mimicked by ATP (2 × 10^−6^ M). The responses consist of a relaxation followed by a “rebound contraction.” Atropine (1.5 × 10^−7^ M), guanethidine (5 × 10^−6^ M), and sodium nitrite (7.2 × 10^−4^ M) were present. Right-hand side: a comparison of the NANC contractile responses of the guinea pig bladder strip to intramural nerve stimulation (NS: 5 Hz, 0.2 ms pulse duration and supramaximal voltage) mimicked by exogenous ATP (8.5 µM). Atropine (1.4 µM) and guanethidine (3.4 µM) were present throughout. (**B**) Effect of changing the calcium ion (Ca^2+^) concentration on the release of ATP (measured with the firefly luciferin/luciferase technique) from the guinea pig isolated bladder strip during stimulation of NANC nerves. Upper trace: mechanical recording of changes in tension (g) during intramural nerve stimulation (NS: 20 Hz, 0.2 ms pulse duration, supramaximal voltage for 20 s). Lower trace: concentration of ATP in consecutive 20 s fractions of the superfusate. The Ca^2+^ concentration in the superfusate varied as follows: (1) 2.5 mM (normal Krebs); (2) 0.5 mM; (3) 0.25 mM; (4) 2.5 mM. The successive contractions were separated by 60 min intervals as indicated by the breaks in the mechanical trace. Atropine (1.4 µM) and guanethidine (3.4 µM) were present throughout. (**C**) The purinergic neuromuscular transmission hypothesis depicting the synthesis, storage, release, and inactivation of ATP. ATP, stored in vesicles in nerve varicosities, is released by exocytosis to act on postjunctional P2 purinoceptors on smooth muscle. ATP is broken down extracellularly by ATPases and 5′-nucleotidase to adenosine, which is taken up by varicosities to be resynthesized and restored in vesicles. If adenosine is broken down further by adenosine deaminase to inosine, it is removed by the circulation. Figure is reproduced with permission from Burnstock and Verkhratsky.^23^

The emergence of ATP and purinergic neurotransmission began in the 1960s when Geoff Burnstock (who was in those days a Senior Lecturer and then Professor of Zoology at the University of Melbourne) discovered and characterized a nonadrenergic, noncholinergic (NANC) neurotransmission in the peripheral nervous system (Figure 2).^19–21^ First, in 1966, Geoff (together with Anne Smythe) performed experiments inspired by Otto Loewy’s seminal study; but the results were only published many years later, in 2010).^22^ Geoff and Anne demonstrated that stimulation of NANC nerves to the taenia coli fixed in a top chamber produced the typical nerve-mediated response (fast relaxation, followed by rebound contraction), while the perfusate from this muscle produced a slower relaxation (without rebound contraction) when being fed into a taenia coli preparation in the lower chamber (Figure 3). Years later, it appeared that the response in the top chamber was driven by ATP, while the response in the lower chamber was mediated by adenosine, which was produced from ATP by a rapid breakdown catalyzed by ectonucleotidases. The realization that the principal chemical transmitter responsible for the NANC is ATP came in 1970, after a series of brilliant experiments on Auerbach’s plexus from turkey gizzard. These experiments demonstrated that ATP (as well as ADP and AMP) was secreted in response to stimulation, which caused inhibition very similar to that triggered by stimulation of NANC nerves. Subsequently, ATP was degraded to adenosine, inosine, and adenine (Figure 4).^24^ Thus, in a single stroke, the fundamental criteria defining ATP as neurotransmitter had been fulfilled. The fundamental criteria are: (1) the criterion of the inactivating enzyme; (2) the criterion of the presence of the transmitter; (3) the criterion of collectability of the transmitter; (4) the criterion of the synthesizing enzyme. Two criteria will be mentioned only en passant, in relation to other criteria: (5) the criterion of the presence of precursors; (6) the criterion of a specific release mechanism. Finally, the fundamental criterion will be discussed, followed by a special case: (7) the criterion of identical actions; (8) the criterion of pharmacological identity.^25^

The theory of purinergic transmission was finally conceptualized in 1972 in one of the most famous (over 1600 citations) papers published in *Pharmacological Reviews.*^26^ This theory was not immediately accepted, the academic community was enchanted by ATP’s role as an energy source; and this spell precluded the realization that so precious a molecule can be wasted by secretion. Of course, at that time, the concept that cells could use a “primitive” molecule involved in metabolism and energy production for highly specific and “noble” functions such as cell-to-cell communication was far beyond anyone’s understanding. Nor was it suspected that the amount of ATP utilized for secretion and extracellular signaling only accounted for an infinitesimal part of the total cellular ATP pool.

The concept of ATP also being used a transmitter even inspired poems, like the one written by Prof. Samuel C. Silverstein in honour of Geoff Burnstock:

Oh tell me Lord how could be,

That though our cells make ATP

It’s not all used for energy,

But sometimes is secreted free.

It puzzles you, it puzzles me

While Geoffrey Burnstock smiles with glee

At the many roles of ATP.

Geoff’s intuition, *in tempore non suspecto*, of the dual role of ATP was absolutely unconventional and “out of the box”. Geoff used to say that if you want to do good science, you have to be “anarchic,” and that to advance knowledge you need to break dogmas. When Geoff was a PhD student at University College London (UCL) together with his best life-long friend and loyal opponent Eric Barnard, they were both part of a Society called “The Challengers” formed by young UCL students who used to meet one night per week to discuss how to break a science dogma.

It took almost quarter of a century for purinergic neurotransmission to become universally accepted. Geoff led this fight and inspired cohorts of followers all over the world, thus finally breaking the resistance and justifying purines as widespread neurotransmitters.

## The Concept of Cotransmission

**Figure 5.**
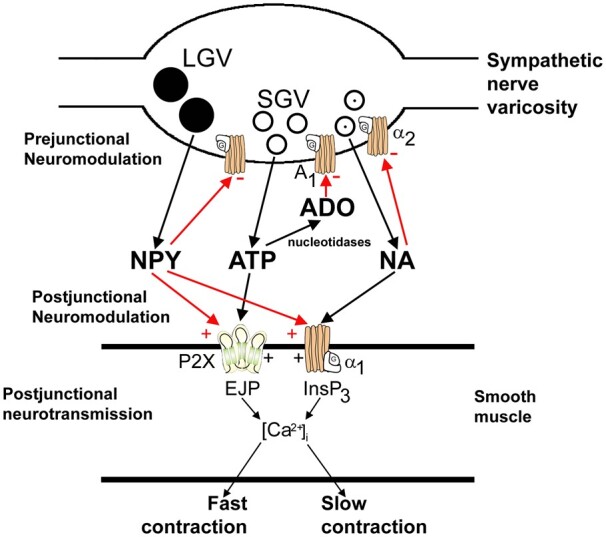
Schematic of Sympathetic Cotransmission as Drawn by Geoff Burnstock. ATP and noradrenaline (NA) released from small granular vesicles (SGVs) act on P2X and α_1_-receptors on smooth muscle, respectively. ATP acting on ionotropic P2X receptors evokes excitatory junction potentials (EJPs), increase in intracellular calcium ([Ca^2+^]_i_), and fast contraction; while occupation of metabotropic α_1_-adrenoceptors leads to production of inositol trisphosphate (IP_3_), increase in [Ca^2+^]_i_ and slow contraction. Neuropeptide Y (NPY) stored in large granular vesicles (LGVs) acts after release both as a prejunctional inhibitory modulator of release of ATP and NA and as a postjunctional modulatory potentiator of the actions of ATP and NA. Soluble nucleotidases are released from nerve varicosities and are also present as ectonucleotidases. Redrawn from Burnstock^26^ and reproduced from Burnstock and Verkhratsky.^27^

Another fundamental contribution of Geoff Burnstock to the general theory of neurotransmission lies in defining cotransmission in both the peripheral and central nervous systems (Figure 5). One of the most long-lasting dogmas of synaptic physiology was the so-called “Dale principle,” the principle postulating that neurons can synthesize, store, and release only a single substance; this was, however, John Eccles^28^ interpretation of Henry Dale lectures and writings, in particular, the 1934 Walter Dixon memorial lecture.^29^ Dale’s statement was that the same neurotransmitter would be stored and released from all terminals of a single neuron. Nonetheless, data started to accumulate demonstrating that neurons are capable of expressing more than one neurotransmitter and again Geoff conceptualized these ideas in a commentary published in *Neuroscience.*^1^ The concept of cotransmission flourished and a wealth of data demonstrated that neurons in both the peripheral and central nervous systems may release more than one neurotransmitter; neurotransmitters can be costored in the same vesicles or dwell in separate vesicular pools that share the same neuronal terminal.^30–36^

## Purinoceptors: The P1/P2 and P2X/P2Y Divide

The difference in the biological actions of ATP and adenosine was noted quite early; Gillespie reported, already in 1934,^37^ that ATP is most potent in relaxing guinea pig ileum, whereas adenosine is more potent in promoting vasodilatation of coronary arteries. Many more examples of pharmacological differences between ATP and adenosine have been since accumulated. Analysis of these differences prompted Geoff to invent the first grand classification of purinoceptors; he suggested^38^ to divide them all into two major classes of P1 (adenosine and AMP) and P2 (ATP and ADP) receptors. This classification became widely used and popular. Adenosine receptors have been further subclassified into A_1_, A_2A_, A_2B_, and A_3_ receptors.^39–41^ The P2 ATP receptors were also in need of stratification, as ATP on its own clearly demonstrated both ionotropic and metabotropic capabilities and in 1985 Geoff (together with Charles Kennedy^42^) classified P2 receptors into P2X and P2Y. This classification was further refined in 1994^43^ when P2X receptors were defined as belonging to ionotropic, whereas P2Y to metabotropic families. Recently, the adenine receptors were proposed to constitute a separate family of P20 receptors in rodents.^44^ In the 1990s, molecular cloning identified seven P2X (P2X_1–7_) receptors^45^ and eight (P2Y_1,2,4,6,11,12,13_,_14_) mammalian P2Y receptors.^46^

Geoff together with his friend and colleague Eric Barnard cloned and characterized the first representative of the P2Y receptor family, the P2Y_1_ receptor from an embryonic chick whole brain cDNA library.^47^ Subsequently, in cooperation with John Wood, Geoff participated in cloning and characterization of P2X3 receptor.^48^ This receptor was unique in that ATP application induced through it a rapidly desensitizing ionic current and that its expression was mostly segregated to a subset of sensory neurons of the dorsal root ganglia. After these initial studies, the role of P2 receptors has been widely demonstrated in the physiological regulation of the central and peripheral nervous systems, the cardiovascular, respiratory, and immune systems, as well as in various pathological conditions. As a result, P2 receptors have gained widespread clinical interest with agonists and antagonists currently undergoing clinical trials; these medicines have therapeutic potential for a broad spectrum of diseases, including thrombosis and stroke, dry eye, atherosclerosis, kidney failure, osteoporosis, bladder incontinence, colitis, neurodegenerative diseases, pain, and cancer (for a more detailed description, the reader is referred to a recent review by Burnstock).^49^ Based on the discovery of P2X_3_ receptors, more recently gefapixant, an orally bioavailable and peripherally restricted P2X_3_ receptor antagonist has been synthesized and explored for its therapeutic potentials in humans.^50^ The first two letters (Ge) are an abbreviation of Geoffrey’s name to honor his merits. In late phase clinical trials, this drug was found to be suitable to suppress chronic cough, but unfortunately, it also caused a high prevalence of alterations in taste sensitivity.

## ATP Storage, Release, and Degradation

The criteria for ATP acting as a neurotransmitter highlighted in the seminal paper published by Burnstock et al.^24^ in 1970 include the demonstration of cellular storage and release. Organellar storage of ATP had previously been demonstrated for chromaffin granules,^51^ for noradrenaline-storing granules isolated from splenic nerves,^52^ and granules from blood platelets.^53^ Storage of ATP in electron-lucent synaptic vesicles was demonstrated somewhat later.^54^ Since large granular vesicles were previously found in axonal profiles in the gut, it was obvious to assume that these also function as storage sites for ATP in NANC inhibitory nerves.^24^

Major research lines followed to further elucidate the functional role of ATP in vesicles and granules. The acridine derivative quinacrine (earlier on used for treatment of malaria), which binds ATP could be used to depict cellular storage of ATP using a fluorescence histochemical method.^55^ In their important study on the guinea pig urinary bladder, Burnstock et al.^56^ identified quinacrine-positive cells that were different from those observed with catecholamine fluorescence and cholinesterase histochemistry, providing further support for the concept that the NANC excitatory nerves supplying the guinea pig bladder are purinergic. This was then followed by many studies using quinacrine—also from Geoff Burnstock’s lab—to identify purinergic nerves. Among others, this allowed the demonstration of the coexistence of ATP and nitric oxide (another inhibitory component) in NANC inhibitory neurons.^57^

The molecular analysis of isolated granules and vesicles added a new twist to purinergic signaling. Not only ATP and biogenic amines but also other nucleotides such as ADP, GTP, UTP, and even the dinucleoside polyphosphates Ap_4_A and Ap_5_A—but not nucleosides—were identified in both chromaffin granules and cholinergic vesicles.^58^^,^^59^ These nucleotides were stored in the millimolar range. Concentrations were always highest for ATP reaching estimated values around 500 mM. In accordance with these findings, it could later be shown that—depending on subtype—nucleotide receptors can also respond to ADP, dinucleoside polyphosphates, UTP, UDP, and UDP-glucose.^60^

It thus became clear that the mechanisms of vesicular nucleotide storage and release were of essential relevance for purinergic signaling. These were characterized in detail in the nervous system, where ATP is stored in dedicated vesicles as well as costored with other transmitters such as glutamate and GABA.^33^^,^^61^ Studies initially concentrated on the molecular and functional characterization of vesicular and granular membrane components as well as the kinetics of nucleotide uptake. While it could be shown that nucleotide uptake into noradrenaline-containing storage granules is energized by an ATP-driven electrochemical gradient with a mean substrate concentration for half saturation of the transport system of about 1–2 mM,^58^ the molecular identity of the transporter remained elusive. In 2008, the vesicular nucleotide transporter (VNUT) was finally cloned and characterized.^62^ This turned out to be a milestone in purinergic signaling in several ways.^63^ The SLC17A9 gene encoding VNUT is present in all animals, further underlining the ubiquity of purinergic mechanisms in the animal kingdom. Similarly, VNUT is widely distributed throughout the body and cellular systems in accordance with the wide distribution of nucleotide receptors and nucleotide signaling pathways in essentially all principal cell types. Moreover, VNUT colocalizes with the vesicular glutamate, GABA, and acetylcholine transporters VGLUT1, VGAT, and VAChT, respectively, and is expressed in tyrosine hydroxylase-positive dopaminergic neurons of the substantia nigra and ventral tegmental area, and in subpopulations of rat dorsal root ganglion neurons. All this beautifully underlines the earlier notion of Geoff Burnstock that ATP acts as a cotransmitter with classical neurotransmitters.^1^ Finally, in VNUT gene knockout mice vesicular ATP release was shown to be blocked in various cellular systems, including neurons and chromaffin cells underlining the important role of exocytosis for cellular ATP release.

In their seminal 1970 paper, Burnstock et al.^24^ demonstrated release of ATP from nonadrenergic inhibitory nerves of the gut. It had previously been shown that ATP can be released from antidromically stimulated sensory nerves^64^ and also from the adrenal medulla.^65^ Yet, while vesicular or granular release was a likely source, it soon turned out that ATP can also be released from non-neuronal cells and by additional mechanisms not involving stored ATP. This further nourished the skepticism of many scientists that ATP release is an artifact and represents leakage from broken or dying cells. The large and negatively charged ATP molecules cannot diffuse through a lipid bilayer. Research of the past two decades has established that cellular release of ATP is far more complicated and that—depending on cell type—several mechanisms are employed. The particular mechanism of ATP release is associated with membrane channels with anion permeability and a large ion-conducting pore. Several molecular complexes have been discussed and are now acknowledged to mediate the physiological release of cytosolic ATP. These include connexin hemichannels, pannexin 1, calcium homeostasis modulator 1 (CALHM1), volume-regulated anion channels (VRACs, also known as volume-sensitive outwardly rectifying anion channels), and maxi-anion channels.^66^ Another promising candidate for an ATP release channel is the P2X_7_ receptor complex.^67^ Thus, the issue is far more complicated than initially anticipated and adds a further puzzle to be solved in the purinergic signaling field.

Extracellular hydrolysis of ATP had been shown early on with spermatozoa or blood cells and in many preparations, the breakdown of released ATP to adenosine could be observed.^68^ Breakdown of ATP had also been observed by Burnstock et al.^24^ in chicken stomach preparations. Accordingly, Geoff Burnstock had laid out the entire extracellular hydrolysis chain from released ATP via ADP and AMP to adenosine followed by reuptake of the nucleoside for resynthesis of ATP and vesicular reloading (see Figure 5).^26^ While this was extremely imaginative, the molecular players involved in extracellular nucleotide hydrolysis were still enigmatic. The first ectonucleotidase cloned and characterized in molecular terms was the tissue nonspecific form of alkaline phosphatase followed by ecto-5′nucleotidase and finally an ecto-apyrase, which was later renamed ecto-nucleoside triphosphate diphosphohydrolase 1 (NTPDase-1).^68^ Surprisingly, it turned out that there was an entire protein family of NTPDases (eight paralogues) with differing hydrolysis profiles and even more groups of enzymes for hydrolysis of ATP (ecto-nucleotide pyrophosphate/phosphodiesterases, NPP1 to NPP4), several alkaline phosphatases were identified and there were AMP hydrolyzing enzymes other than ecto-5′-nucletidase (alkaline phosphatase, prostatic acid phosphatase). There is also interconversion leading to the extracellular formation of ATP from its breakdown products. Finally, some of these enzymes also occur in soluble form.^69^ The differential cellular distribution of these enzymes and their catalytic properties provides a further challenge to understanding the physiology and pathophysiology of purinergic signaling.

## Ancient and Ubiquitous Purinergic Transmission

Geoff was very much interested in the evolution of purinergic transmission.^23^^,^^70–72^ He clearly recognized and described a long evolutionary history of purinergic transmission, and the tight links with the fundamentals of the genetic code and bioenergetics. Geoff proposed that ATP could have been the “first” molecule during evolution to communicate messages to other cells. His idea was based, first, on the fact that any kind of cell does synthesize and utilize ATP, and, second, that P2X receptors share topological membrane organization with epithelial voltage-gated sodium channels. Geoff used to say that when, during evolution, cells experienced the need to send a message to other cells, they “decided” to secrete something that was present in huge amounts inside the cells themselves—that is ATP. To communicate its message to nearby cells, secreted ATP “induced” some already existing entity that was present on the extracellular membrane of nearby cells to recognize itself as a message. Primitive voltage-gated epithelial sodium channels were thus induced to specialize themselves to recognize ATP. Geoff used to conclude that this could have been how voltage-gated channels had turned into ligand-operated receptors. These views were presented by Geoff at a Ciba Foundation meeting in London in 2005, thus instigating an exciting, smart, and exhilarating discussion between Geoff and Eric Barnard, whose comment to Geoff ‘s theory was “If I were a neurone, I would not use ATP as a transmitter.”

Geoff also very clearly perceived the omnipresence of purinergic transmission widespread between species. Bacteria, although not possessing a classical purinoceptor are sensitive to ATP which acts as a danger signal; the *Dictyostelium discoideum* is the earliest species to express ionotropic P2X-like *Dd2PX* receptor^73–75^ and metabotropic receptors to cAMP, known as cAR1-4 receptors, which are ancestors of P2Y receptors.^76^ Purinergic transmission is present in algae, in plants, in fungi and throughout the animal kingdom.^23^ Furthermore, purinergic transmission is unique in its pluripotency and omnipresence. Most neurotransmitters are segregated around the body: for example, glutamate and GABA are operative in the brain, glycine is the main inhibitory transmitter in the spinal cord. Acetylcholine has a wider spread being deployed not only in the peripheral nervous system and in a subpopulation of cholinergic neurons in the brain, but acting as a transmitter in other organs. Adrenaline and noradrenaline have a somewhat wider reach acting as central neurotransmitters and circulating hormones. The purinergic system, has no boundaries acting in all organs, tissues, and systems, mediating rapid as well as long-lasting effects^77^ because almost every cell does have at least one type of purinoceptors. Most fundamental physiological functions such as sensory transduction, regulation of heart rate, smooth muscle contraction, bile secretion, endocrine regulation, immune responses, as well as various pathophysiological conditions, including neuropathic pain, diabetes, kidney failure, and cancer, are regulated by purinergic signaling.^72^^,^^78^ Finally, activation of P2X_7_ receptors signals the very beginning of life: using this pathway ATP induces the acrosome reaction which is needed for sperm to fuse with the oocyte plasma membrane and subsequent successful fertilization.^79–81^

## Purinergic Signaling in the Central Nervous System

After discovering the role of ATP as an extracellular signaling molecule in the peripheral nervous system, it was only natural that Geoff should turn his attention to the brain. Frances Edwards et al. were the first to describe the role of ATP as a rapid neurotransmitter in the medial habenula^82^; subsequently numerous studies characterized ATP as a fast neurotransmitter in several brain regions in various areas of the brain contributing to fast neuro-neuronal synaptic communication.^83^^,^^84^ The noradrenergic cells of the nucleus locus coeruleus were found to exhibit P2X receptor-mediated excitatory postsynaptic currents (EPSCs) and α_2_-adrenoceptor-mediated inhibitory postsynaptic currents (IPSCs), indicating cotransmission mediated by ATP and noradrenaline.^85^ However, when the brain and spinal cord were electrophysiologically mapped in search for ATP-mediated fast neurotransmission, it turned out that relatively few areas utilize fast ATP signaling via P2X receptors. Furthermore, ATP-mediated ECSCs caused by stimulation of postsynaptic P2X receptors were relatively small, infrequent, only observed in a subpopulation of neurons, and strong electrical stimulation was required to evoke those.^86^

**Figure 6.**
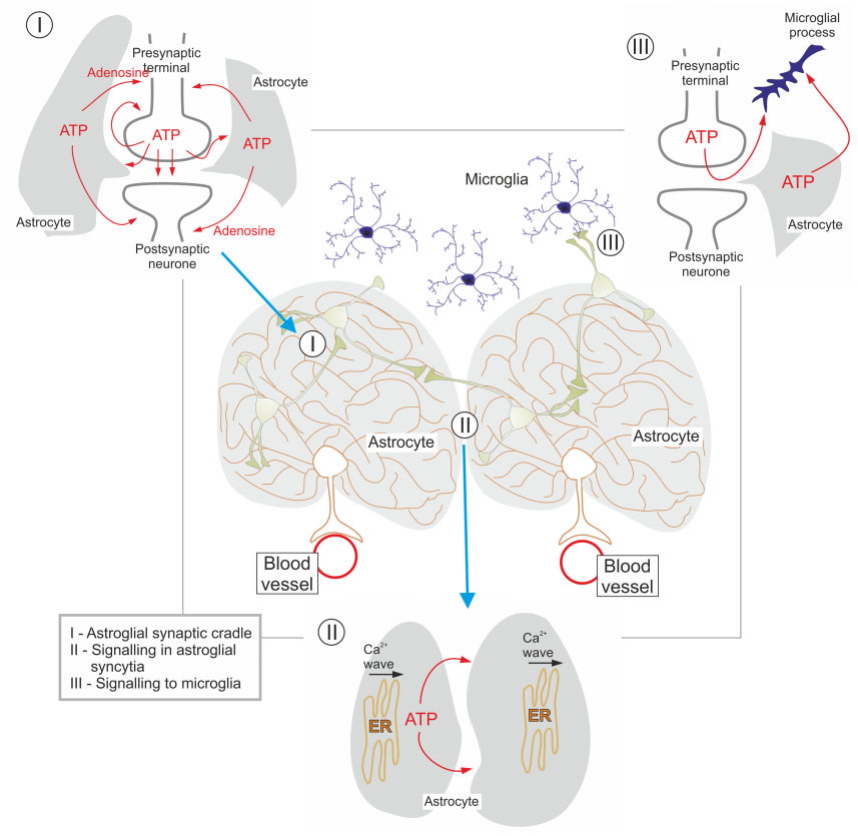
Omnipresence of Purinergic Signaling Pathways in Neuronal–Glial Circuits. The microarchitecture of the gray matter (as shown in the center) is defined by astroglial domains, composed of astrocyte, neighboring blood vessel encompassed by astroglial end feet and neurons residing within astroglial territory—the neuro-glio-vascular unit. The microglial cells (each also having its own territory) are constantly surveying these domains spying for damage. ATP and its derivatives act as an extracellular signaling molecule at all levels of communications within neuronal–glial networks. Within the astroglial synaptic cradle^88^ formed by perisynaptic astrocytic processes (I), ATP, released during synaptic transmission, activates astrocytic receptors, which in turn initiate Ca^2+^ signals and Ca^2+^ waves in astroglial syncytia. Astroglial is capable of releasing ATP, which feeds back to neurons via activation of pre- and postsynaptic P1 and P2 receptors. ATP released from astrocytes (II) triggers and maintains astroglial Ca^2+^ waves. Finally, ATP released from all types of neural cells control activation (III) of microglia. Redrawn and modified from Verkhratsky et al.^89^

It was an important discovery that ATP is released by exocytosis from neurons and neuroglia, although the transporter-mediated and diffusion/channel-mediated release of ATP from glial cells is probably of equal significance. The different temporal resolution of purinergic signaling is of utmost significance; while P2X receptors mediate fast responses in the millisecond-range, P2Y receptors mediate responses in the range of seconds, hours, or even days/weeks by interacting with cytoplasmic signaling cascades and growth factors.^87^ In addition, ATP released by neurons may activate glial P2X/Y receptors and vice versa, ATP secreted from neuroglial cells may functionally feed-back to neuronal purinoceptors (Figure 6).^89^ Purinergic signaling, therefore, appears as the main pathway for neuronal–glial reciprocal communications, while ATP secreted by both neurons and neuroglia contributes to the regulation of various aspects of neuroplasticity.^90^

Geoff never forgot that basic science provides the background on which applied science is building up. He consequently looked for the applicability of his findings for the benefit of patients. While studying the role of ATP in neuron–neuron and neuron–glial communications, he always wanted to decipher the behavioral consequences of this interaction. For example, in 2011, he found that astrocytic ATP is able to modulate memory in chicks.^91^ This was an important extension of the previous findings that purinergic pathways are related to feeding behavior, locomotor coordination, sleep, and arousal, as well a mood and motivation.^92^

Geoff’s primary interest in the last decades in his life was in neurodegeneration and regeneration of nervous tissue. He wrote many conceptual review articles on the involvement of ATP in brain injury, stroke, ischemia, epilepsy, chronic pain, Alzheimer’s disease, Parkinson’s disease, multiple sclerosis, and amyotrophic lateral sclerosis.^92^^,^^93^ He found it fascinating that ATP and its receptors appear to be major players in the etiology of psychiatric disorders such as major depression, bipolar disorder, autism, and addiction. With respect to neuroregeneration, he identified P2 receptors whose blockade inhibited the injury-induced proliferation at olfactory epithelium progenitor cells of the adult mouse.^94^ This was in excellent agreement with the localization of a plethora of P2 receptors at embryonic and adult neural progenitor cells in the rodent and human brain.^95^

Geoff was not only a most original scientist, who made many seminal discoveries but he continuously developed, based on these discoveries, the most inspiring hypotheses, which continuously fertilized the field of purinergic signaling. Here it is probably worth referring to a minor touch of Geoff’s genius that influences purinergic research. In 2009, he proposed a hypothesis for the purinergic basis of acupuncture.^96^ This stirred the field and initiated quests for purines and purinoceptors in acupuncture studies.^97^^,^^98^

## Purinergic Signaling Extends into Pathology

We were all taught in Medical School that tissue damage triggers inflammation with the associated canonical signs: *Rubor, Tumor, Calor, Dolor* (Aulus Cornelius Celsus, *De Medicina*) with Galen’s addition of *Functio laesa*. It has always been clear to every pathologist that injured tissues release factors that are able to signal damage, alert resident immune cells, start and amplify inflammation. Many investigators have been long intrigued by the molecular identity of these hypothetical “alarm factors,” even more so after we all became aware that cells might also die by a “silent” death (apoptosis) that, as opposed to necrosis, minimizes stimulation of the inflammatory system.

We now know that the most powerful and ubiquitous “alarm factor” (or damage-associated molecular pattern (DAMP)) released from any injured tissue is ATP, nothing else than adenosine 5′-triphosphate.^99^ With hindsight, this seems all too obvious, given the biochemical properties of ATP and the large ATP concentration gradient between the interior of the cell and the extracellular space. However, it is only after Geoff Burnstock put forward his hypothesis on the extracellular signaling role of ATP that biologists, immunologists, pathologists, and all other life science students started to realize that ATP is much more than the universal biological energy currency.

## ATP: The Perfect Extracellular Danger Messenger

The concept that ATP is an extracellular messenger, and a signal of danger, was so obvious (maybe too obvious!!) that paradoxically it was resisted by most investigators. Yet, ATP has all the key features of the perfect DAMP: basically nil extracellular concentration versus very high intracellular level, high mobility in the aqueous extracellular milieu, a very efficient extracellular degrading system to prevent desensitization, specific plasma membrane receptors. Another serious objection was raised to question the validity of the purinergic hypothesis outside the nervous system: there is no evidence that ATP accumulates to any extent in non-neuronal tissue. This was a considerable obstacle to the acceptance of an extracellular signaling role of ATP. There was of course *in vitro* evidence that many different cell types released ATP via multiple pathways (ABC transporters, connexin-43, pannexin-1, secretory vesicles), but no solid experimental proof that this might occur *in vivo*. The turning point was the introduction of a genetically encoded bioluminescent probe (the pmeLUC probe) that allowed semiquantitative *in vivo* measurement of the ATP concentration in the pericellular space.^100^ Thanks to the pmeLUC probe we now know that ATP accumulates into the extracellular space during bacterial infections, allogeneic reactions, autoimmune reactions, traumas, and of course at tumor sites.

## ATP and Purinergic Receptors: Partners in Inflammation

Of course, any DAMP, and ATP is no exception, is by definition a potent proinflammatory stimulus. Ample experimental evidence supports the original view that a host of injurious agents and pathogens trigger ATP release from virtually all cell types. Released ATP in turn acts as an autoparacrine stimulus to activate inflammatory cells through P2Y and P2X purinoceptors. The P2Y/P2X receptor classification^42^^,^^43^ found a fertile application to inflammation. It was soon realized that all immune cells express P2Y and P2X receptors, a feature that confers on the immune response an enormous plasticity. P2Y and P2X receptors are involved in inflammatory pain, inflammatory edema, release of inflammatory mediators, production of reactive oxygen and nitrogen species, chemotaxis, proliferation, differentiation, immune cell metabolism, necrosis, necroptosis, and apoptosis.^101^ A paradigmatic example is the P2X_7_ receptor that is now recognized to be one of the most potent stimulants of the NLRP3 inflammasome and of interleukin-1β release, and therefore a key trigger of inflammation.

## ATP and Purinergic Receptors Are Key to Inflammatory Homeostasis and in Host–Tumor Interaction

Inflammation is a tightly controlled homeostatic system. Any homeostatic system is based on feed-forward and feed-back control mechanisms working in close association. Geoff Burnstock’s discovery of the extracellular role of ATP paved the way for a thorough understanding of the role of extracellular adenosine in immunity and inflammation. The large majority of adenosine is generated in the extracellular space at the expense of ATP thanks to powerful and ubiquitous ectonucleotidases, for example, CD39 and CD73.^102^ One of the most important breakthroughs in recent years has been the discovery of the immunosuppressant function of adenosine.^103^ This discovery has far-reaching implications in immunology and more importantly in cancer. It is now an established fact that the tumor microenvironment (TME) is highly immunosuppressive due to the accumulation of agents that inhibit antitumor T and NK lymphocyte responses. One of the most potent immunosuppressants in the TME is adenosine generated by the hydrolysis of ATP that accumulates to very high levels in this restricted environment.^104^ On the contrary, extracellular ATP itself in the TME has multiple roles, tumor-promoting, by supporting tumor cell proliferation, or tumor-suppressing, by fuelling the anticancer immune response. Again, differential expression of P2Y and P2X receptors by tumor cells and tumor-infiltrating inflammatory cells confers an extraordinary plasticity to purinergic signaling in cancer. One of the most remarkable consequences has been the application of this knowledge to cancer therapy: over 50 clinical studies are ongoing to test the effectiveness of adenosine receptor blockade in different types of cancer. This would not have been possible without Geoff Burnstock’s intuition of the extracellular messenger role of ATP.

As Geoff Burnstock might reply to those who belittled his prophetic vision of the role of extracellular ATP: “There are more things in heaven and earth, Horatio, than are dreamt of in your philosophy.” This is what we learned from Geoff: there is more in life and science than we ever dreamt.

## Authors’ Contribution

All authors contributed equally to this work

## Conflict of interest statement

The authors have declared that no conflict of interest exists.
